# SUBJECTIVE VIEWS OF AGING ARE ALL AROUND: A SOCIAL PERSPECTIVE

**DOI:** 10.1093/geroni/igae098.0547

**Published:** 2024-12-31

**Authors:** Yaakov Hoffman, Amit Shrira, Shevaun Neupert

**Affiliations:** Chair; Co-Chair; Discussant

## Abstract

Subjective Views of Aging (VoA) of the individual older adult are important predictors of many health and wellbeing outcomes. Nevertheless, VoA often develop within a relational context, suggesting that VoA have an inherent interpersonal component. However, few works have considered VoA within the greater social perspective. In this session we address VoA in two different social contexts; namely medical situations, and marital context. We address how these wider social VoA impact older adults’ health and wellbeing. The first talk (Shrira & Shafir) addresses how ageist attitudes of spousal caregivers impact rehabilitation outcomes in patients following osteoporosis fractures. In the second talk (Hoffman et al.), we address how ageist attitudes of medical staff impact older adult patients in internal wards, leading to negative VoA, and to deteriorated physical health months later. The third talk (Mukherjee & Neupert) addresses increased daily memory failures of older adults with arthritis following exposure to interpersonal stressors, when awareness of age-related losses is low. The fourth talk (Bodner, Segel-Karpas & Estlein) explores how couples experience their joint aging, introduces a new questionnaire, and shows how this measure correlates with marital satisfaction and other variables. Together, the findings highlight how VoA operate within social contexts and offer valuable information for both researchers and clinicians regarding VoA and their effects on health and wellbeing.

